# A Public Data Set of Videos, Inertial Measurement Unit, and Clinical Scales of Freezing of Gait in Individuals With Parkinson's Disease During a Turning-In-Place Task

**DOI:** 10.3389/fnins.2022.832463

**Published:** 2022-02-23

**Authors:** Caroline Ribeiro De Souza, Runfeng Miao, Júlia Ávila De Oliveira, Andrea Cristina De Lima-Pardini, Débora Fragoso De Campos, Carla Silva-Batista, Luis Teixeira, Solaiman Shokur, Bouri Mohamed, Daniel Boari Coelho

**Affiliations:** ^1^Human Motor Systems Laboratory, School of Physical Education and Sport, University of São Paulo, São Paulo, Brazil; ^2^BIOROB Laboratory, Ecole Polytechnique Fédérale de Lausanne, Lausanne, Switzerland; ^3^Laboratory of Integrative Motor Behaviour, Centre for Neuroscience Studies, Queen's University, Kingston, ON, Canada; ^4^Center for Mathematics, Computation, and Cognition, Federal University of ABC, São Bernardo do Campo, Brazil; ^5^Exercise Neuroscience Research Group, School of Arts, Sciences, and Humanities, University of São Paulo, São Paulo, Brazil; ^6^Department of Excellence in Robotics and AI, The BioRobotics Institute, Scuola Superiore Sant'Anna, Pisa, Italy; ^7^Biomedical Engineering, Federal University of ABC, São Paulo, Brazil

**Keywords:** biomechanics, motion analysis, wearable sensors, kinematics, movement disorders

## Introduction

Freezing of gait (FoG) is a widespread Parkinson's disease (PD) symptom. It is a disabling, complex, and highly variable clinical phenomenon characterized by brief episodes of inability to step or very short steps (Nutt et al., 2011). This condition usually leads to falls (Paul et al., 2013), falls-related injury, and ultimately reduced independence in the activities of daily living (Gilat et al., 2018). FoG is often described as the sensation of having the feet glued to the floor while the body center of mass continues to move forward. There is a growing interest in non-pharmacological interventions to manage FoG, and reliable tools are required to determine the severity of this symptom. Wearable sensors are emerging as new tools to obtain information about FoG objectively. A typical wearable sensor consists of a microelectromechanical system with a gyroscope (angular position rate sensor), an accelerometer (acceleration sensor), and a magnetometer (orientation sensor) with one, two, or three axes at each sensor. Several automatic freeze-detection algorithms based on some or all signals of one or multiple wearable sensors have been developed (Silva De Lima et al., 2017; Pardoel et al., 2019). These methods vary in complexity, from frequency domain analysis (Moore et al., 2008, 2013; Delval et al., 2010; Capecci et al., 2016), wavelet transforms (Punin et al., 2019) to multiple features and machine-learning techniques (Tripoliti et al., 2013; Ahlrichs et al., 2016).

A major limitation to developing robust algorithmic methods for FoG estimation is the lack of a large public data set presenting kinematic data recorded on PD patients. To the best of our knowledge, there are four open data sets^1^ (Bachlin et al., 2010; Mazilu et al., 2013; Li, 2021; Pardoel et al., 2021) with some of these characteristics. For example, (a) The Daphnet (Bachlin et al., 2010) contains acceleration data for 10 PD patients in several laboratory walking tasks, and (b) CuPiD (Mazilu et al., 2013) included 35 motion and three physiological sensors in 18 PD patients. Both datasets were recorded during walking and studied FoG features of walking.

Our current work fills the gap in two critical points. First, none of these studies included data during the turning phase; this is an important limitation as studies have shown that FoG events are very frequent during the turning phase in PD patients (Mancini et al., 2017). Second, given the variability of the patients' conditions (severity of the PD, level of medication), precise control of the clinical and medication status is necessary. Since individuals remain ON medication for most of the day, the dataset should include measurements in this medication condition (the Daphnet presents data during OFF medication condition, and no information is provided in CuPiD).

Here we present an open data set of PD patients, which includes both (a) study patients' demography, (b) clinical conditions (PD severity, the number and duration of FoG episodes for each individual, clinical scales, and medication state during testing), and kinematics (video, acceleration, and angular velocity) during a turning-in-place task in individuals with PD in the ON medication state.

## Methods

The data collection was performed in the School of Physical Education and Sport at the University of São Paulo, Brazil. The local ethics committee approved this study, and all patients signed a consent form before the data collection. As FoG is sporadic, the PD patients participated in three experimental sessions at an interval of 1 month between each session to increase the chances of occurrence. Since medication can influence the presence of the FoG and that most PD subjects are ON medication, we performed our measurement during this ON medication condition, meaning a stable dose of antiparkinsonian medication for at least 1 month, and they had taken dopaminergic medication 1 h before starting the sessions to ensure dose stabilization.

### Participants

A convenience sample of 35 idiopathic PD patients with FoG (16 females and 19 males) was recruited to participate in this study. The patients were recruited from the Movement Disorders Clinic in the School of Medicine at the University of São Paulo. According to the UK Brain Bank criteria, the diagnosis was confirmed by a movement disorders specialist. The patients were interviewed to collect their demographic, socio-cultural, and overall health conditions. Their ages varied from 44 to 84 years, body masses from 47.0 to 100.0 kg, heights from 1.46 to 1.89 m, Hoehn and Yahr (H&Y) scale between 2 and 4. Inclusion criteria were the absence of neurological or physical dysfunctions other than those associated with PD, no surgery for PD, and no diagnosed vestibular, visual, or somatosensory dysfunctions as self-declared, and patients should be able to walk independently. The ethics committee from the School of Physical Education and Sport at the University of São Paulo approved the study protocol.

### Procedures

The following data-collection procedures were implemented. At the first session:

1. The researcher explained to each patient the process of data collection.
2. After these explanations, the patient signed the informed consent form.
3. The researcher interviewed the participants to collect their clinical data, medication, and disease diagnosis.

In each session:

4. Two experienced researchers in movement disorders applied the following scales: Unified Parkinson's Disease Rating Scale motor aspects of experiences of daily living (UPDRS-II) and motor aspects (UPDRS-III), H&Y (Hoehn and Yahr, 2001), New Freezing of Gait Questionnaire (NFoG-Q) (Nieuwboer et al., 2009), Mini Balance Evaluation Systems Tests (mini-BESTest) (Horak et al., 2009), Fall Efficacy Scale-International—FES-I (Yardley et al., 2005), Hospital Anxiety and Depression Scale (HADS) and two subscales, the Hospital Anxiety Scale (HAS) and the Hospital Depression Scale (HDS) (Zigmond and Snaith, 1983), Mini-Mental State Exam score (MMSE) (Folstein et al., 1975), Montreal Cognitive Assessment (MoCA) (Nasreddine et al., 2005), and Frontal Assessment Battery (FAB) (Dubois et al., 2000). The assessments of each item on the scales were given by consensus among researchers.
5. Participants rested for 10 min.
6. All trials were performed barefoot, and the participants wore comfortable clothes. Participants made three trials of the turning in place while wearing an inertial measurement unit (Physilog 5 by Gait Up, CH) on the shank of the most affected body side. An inertial measurement unit (IMU) consists of a microelectromechanical system with a triaxial gyroscope (angular position rate sensor) and a triaxial accelerometer (acceleration sensor). Participants stood and turned for the turning-in-place task, alternating 360° turns to their right, then 360° to their left, repeating this sequence at a self-selected pace for 2 min. One of the researchers stood near the participant to help in cases of significant disequilibrium when performing the task.

### Data Acquisition and Processing

The inertial sensors recorded triaxial linear accelerations and triaxial angular velocities at 128 Hz. The same axes of orientation of the IMU depended on the subject's leg shape and how they walked. In a standing position with the feet parallel to each other, the y-axis was approximately vertical, and the positive direction pointed downward, the z-axis was approximately horizontal (anteroposterior direction), the positive direction pointed forward, and the x-axis direction was determined by the right-hand rule (mediolateral direction)—a software code for the IMU -managed data acquisition. After the acquisition, the data were uploaded to a computer in a single file for each trial *via* Matlab. Offline, accelerometer, and gyroscope data were low-pass fourth-order zero-phase Butterworth filtered with a 60-Hz cutoff frequency. The beginning of the turn was defined by the moment when the vertical acceleration was higher than 5% of the maximum value.

Turning trials were recorded video through a commercial digital camera (Sony, 30 Hz). The beginning of the turn in the video was defined as the first moment the patient performed an apparent movement in the feet. We consider akinetic FoG, trembling FoG, and festinating FoG the same phenomenon. Two movement disorders specialists reviewed the videos together and noted FoG using the ELAN software (Gilat, 2019). The FoG was discussed and resolved by consensus among researchers. The beginning of the FoG episode was defined when the turn pattern (alternating right and left steps) was arrested or if it appeared as if they were trying unsuccessfully to initiate or continue the turn. The end of an episode was defined as when an effective step had been performed and followed by continuous turning (Mancini et al., 2021).

In addition, one trial was acquired with the subject standing upright and as still as possible for 10 s, in case someone wants to calibrate the sensors.

**Figure 1:**
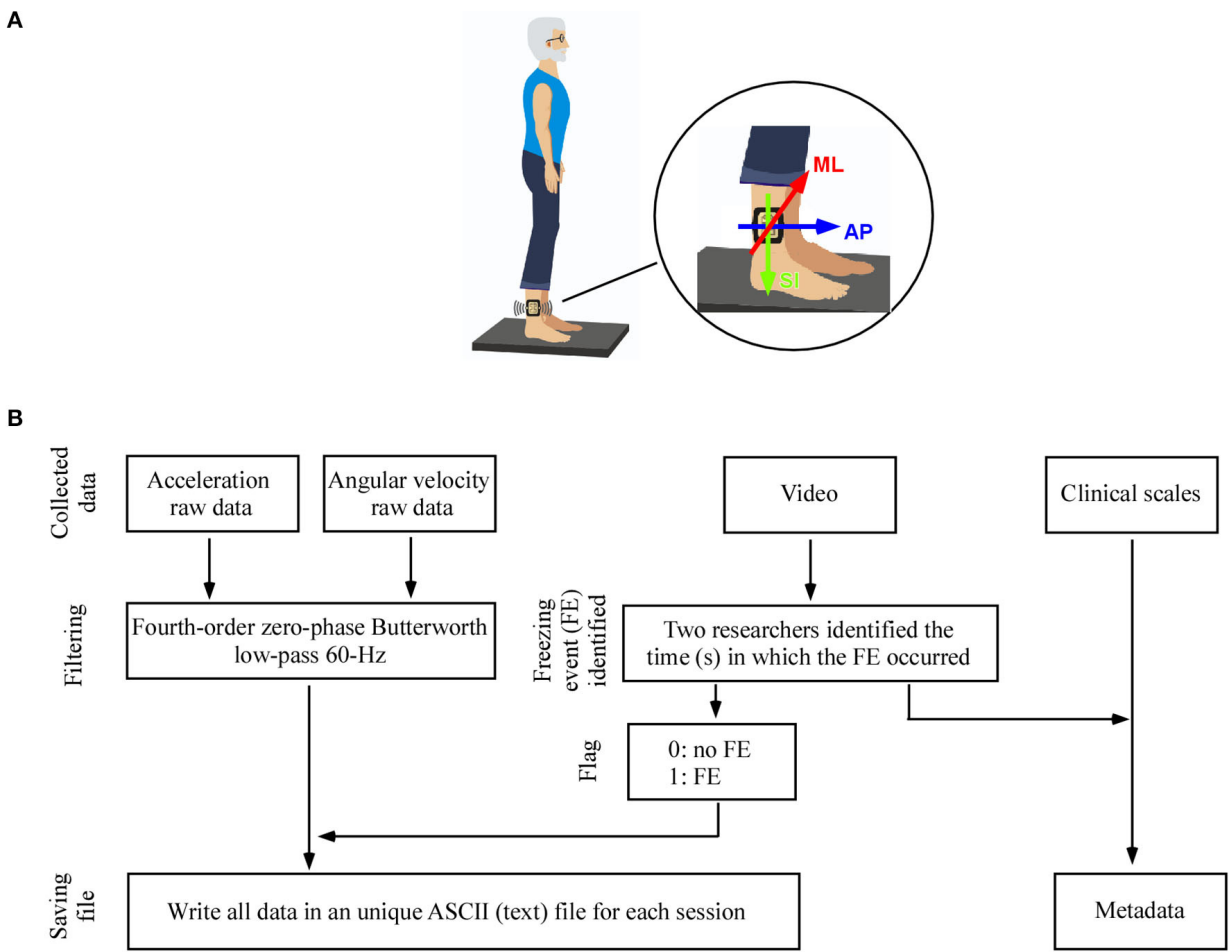
**(A)** Placement of the wearable unit on the participant' leg. In a standing position with the feet parallel to each other, the y-axis was approximately vertical, and the positive direction pointed downward, the z-axis was approximately horizontal and the positive direction pointed forward, and the x-axis direction can be found by the righthand rule. **(B)** Flowchart of signal preprocessing steps applied to the data.

Placement of the IMU and a flowchart with the preprocessing steps are presented in Figure 1.

### Technical Validation

The FoG-ratio has been based on power spectral density from the anteroposterior acceleration data. The FoG-ratio was then calculated as the ratio between the square of the total power in the frequency band corresponding to freezing episodes (3–8 Hz) and the total power in the frequency band corresponding to locomotion (0.5–3 Hz). Thus, higher FoG-ratio scores indicate greater FoG severity (Mancini et al., 2017). Finally, Spearman correlation coefficient (one-tailed) was used to determine the correlation of subjective FoG measures (NFoG-Q score) with the total time of FoG during the turning task (s) and FoG-ratio.

## Results

Participants spent a total of 1,611 s in FoG. On average, participants experienced 3.0 (SD = 2.9) FoG episodes, but 36% did not have FoG episodes during the turning task. The data is available at Figshare (https://doi.org/10.6084/m9.figshare.14984667) under the CC0 4.0 license. The data set contains three types of information: (a) metadata, (b) separate text files were generated for the IMU processed data for each session, and (c) the video of each session. In addition, the clinical characteristics of the patients are presented in Table 1.

**Table 1:** Mean (standard deviation) of the characteristics of the participants.

	**Session 1**	**Session 2**	**Session 3**
Disease duration (years)	8.04 (4.10)		
L-Dopa equivalent units (mg∙day^−1^)	675.21 (277.35)		
NFoG-Q (score)	17.72 (5.63)	13.04 (8.01)	16.21 (6.10)
MiniMental (score)	26.63 (2.72)	27.04 (2.94)	26.86 (3.48)
HandY stage (score)	2.91 (0.51)	2.88 (0.51)	2.86 (0.36)
UPDRS-II (score)	8.54 (3.78)	6.93 (3.13)	7.29 (2.64)
UPDRS-III (score)	31.83 (14.99)	27.81 (12.80)	24.43 (11.57)
HADS-A (score)	9.8 (5.26)	9.48 (5.73)	10.86 (6.11)
HADS-D (score)	8.6 (5.19)	8.41 (5.58)	10.21 (6.27)
Mini-BESTest (score)	18.37 (6.86)	22.41 (5.17)	23.29 (4.32)
FES-I (score)	33.94 (11.58)	30.85 (11.13)	28.43 (11.67)
TUG (s)	14.44 (7.29)	11.32 (3.41)	12.76 (8.15)
TUG dual task (s)	18.87 (13.36)	14.83 (10.31)	15.36 (12.13)

*MoCA, Montreal Cognitive Assessment; HandY, Hoehn and Yahr; NFoG-Q, New Freezing of Gait Questionnaire; UPDRS-II, motor aspects of experiences of daily living Unified Parkinson's disease rating scale; UPDRS-III, motor score Unified Parkinson's disease rating scale; Mini-BESTest, Mini-Test of Balance Assessment System scale; FES-I, Falls Efficacy Scale International; TUG, Timed Up and Go test*.

### Metadata

The metadata file named PDFEinfo.txt contains 61 information from each patient's anamnesis and clinical scales. Here is the coding for the metadata:

1. ID: the file name of the stabilography trial (PDFEXX, where PDFE means Parkinson's disease Freezing Event; XX identifies the patient and varies from 01 to 35).
2. Age: patient's age in years.
3. Height (cm): height in meters (measured with a calibrated stadiometer).
4. Weight (kg): weight in kilograms (measured with a calibrated scale).
5. Gender: gender (F or M).
6. Disease duration (years): year from diagnosis.
7. Handedness: a self-reported manual preference.
8. More affected side: the more affected body side was defined as the side with the highest UPDRS-III score, items 3.3–3.8 and 3.15–3.17.
9. L-Dopa equivalent units (mg•day^−1^): total daily levodopa equivalent dose in mg•day^−1^ according to Tomlinson et al. (2010).
10. Sessions #: number of turning in-place trials performed by the participant.

For each session:

1. Mini-Mental: total score of the MMSE scale.
2. NFoG-Q: total score of the New Freezing of Gait Questionnaire.
3. Hoehn and Yahr: score of H&Y.
4. UPDRS-II: total score of the UPDRS-II.
5. UPDRS-III: total score of the UPDRS-III.
6. PIGD: score of Postural Instability/Gait Difficulty (PIGD), according to Stebbins et al. (2013).
7. Dyskinesia: score of item 15—dyskinesia of UPDRS-III.
8. HADS: total score of the HADS.
9. HADS-A: anxiety subscale of the HADS.
10. HADS-D: depression subscale of the HADS.
11. FES-I: total score of the FES-I.
12. miniBESTest: total score of the miniBESTest.
13. TUG time: time (s) of the Timed Up and Go test (TUG).
14. TUG dual-task time: time (s) of the TUG with dual-task.
15. Time of FoG (s): time(s) the freezing episodes occurred in the session.
16. Total time in FoG (s): total freezing time (s) in session.
17. The “time of FoG (s)” columns are organized as follows: [start time-end time in s (1st FoG); start time-end time (2nd FoG); …].
18. Numbers of FoG episodes (*n*): total numbers of FoG episodes in session.
19. FoG ratio: based on power spectral density from the anteroposterior acceleration data.

### Processed Data

All data set are stored in ASCII (text) format. Each text file with the data is named by the corresponding ID plus the number of sessions. Each file has a header and 15,360 rows (120 s × 128 Hz), and nine columns:

1. Frame: frame number.
2. Time (s): time in s.
3. ACC ML (g): mediolateral accelerometer in units of gravitational acceleration.
4. ACC AP (g): anteroposterior accelerometer in units of gravitational acceleration.
5. ACC SI (g): vertical accelerometer in units of gravitational acceleration.
6. GYR ML (deg/s): mediolateral gyroscope (angular velocity in degrees/s).
7. GYR AP (deg/s): anteroposterior gyroscope (angular velocity in degrees/s).
8. GYR SI (deg/s): vertical gyroscope (angular velocity in degrees/s).
9. Freezing event (flag): from the identification of FoG carried out by the movement disorders specialists using the video; we identified when these events happened in the IMU data (0 for no-FoG and 1 for FoG).

### Video

Each video file is named by the corresponding ID plus the number of sessions. We only filmed the patients' lower limbs.

### Technical Validation

There was a small but statistically significant correlation between NFoG-Q score and FoG-ratio (rho = 0.39, *p* < 0.001). The correlation between total time of FoG during turning task and NFoG-Q score (rho = 0.56, *p* < 0.001) and FoG-ratio (rho = 0.63, *p* < 0.001) were significant.

## Discussion

We presented an open data set of acceleration and angular velocity data from wearable sensors placed on one leg, the video during the turning task, plus a file with metadata containing clinical information in individuals with PD. In addition, data were generated from the evaluation of 35 PD patients with FoG. Furthermore, the open data set includes the identification of FoG carried out by movement disorders specialists using videos for qualitative analysis.

Our results demonstrated a significant correlation between subjective (NFoG-Q) and objective (FoG-ratio, and total time of FoG during turning task) FoG measures, as observed previously (Mancini et al., 2017). This result is interesting for two reasons. First, NFoG-Q is related to a patient's perception of FoG duration and frequency that they had experienced during the past month; however, it is subjective and may represent a weak indication of FoG severity out of the stimulating clinic environment (Mancini et al., 2019). Second, despite the turning task being an objective lab-based method to measure freezing severity without considering the patient's self-perception, FoG episodes may disappear during the lab examination due to the patient paying extra attention to gait (Mancini et al., 2019). Even so, the finding that both FoG measures are associated confirms that the patients' judgment of their freezing severity was associated with FoG severity assessed by an objective lab-based method.

To the best of our knowledge, this is the first data set that includes, in addition to kinematic data, cognitive or psychiatric scales in patients with PD and FoG. These scales are extremely important when studying FoG. For example, anxiety was the strongest predictor of FOG development after 15 months of the initial diagnosis (Ehgoetz Martens et al., 2018). Thus, anxiety may act as a stressor and trigger FOG in daily activities, as FOG occurs at home (Mancini et al., 2019), and patients may have feelings of inability to perform any activities due to lack of confidence and fear of falling.

Previous FoG datasets with wearable sensors have been published elsewhere (Bachlin et al., 2010; Mazilu et al., 2013). Although these studies presented valuable information, the open data set we showed in this study is unique in the literature, given the medication status and a complete description of the clinical status of the participants. One exciting and promising future step for researchers could be developing new FoG detection algorithms that contain the patients' clinical and physiological information. Despite the patients being classified as FoG, 36% did not have FoG episodes during the turning task. This information could be used as a control in identifying patterns of FoG episodes. A limitation of our data set is the use of only one sensor; studies have shown that multi-sensor fusion systems can perform better than single-sensor systems (Bachlin et al., 2010; Li et al., 2020). Nevertheless, our data set will be interesting for the researcher to develop decoding algorithms with minimal hardware.

## Data Availability Statement

The datasets presented in this study can be found in online repositories. The names of the repository/repositories and accession number(s) can be found below: 10.6084/m9.figshare.14984667.

## Ethics Statement

The studies involving human participants were reviewed and approved by Federal University of ABC. The patients/participants provided their written informed consent to participate in this study. Written informed consent was obtained from the individual(s) for the publication of any potentially identifiable images or data included in this article.

## Author Contributions

CR: conceptualization, methodology, data curation, and writing—original draft. RM and JÁ: methodology and data curation. AL-P and DF: writing—original draft. CS-B and LT: writing—review and editing. SS: writing—original draft, writing—review, and editing. BM: project administration and funding acquisition. DC: conceptualization, methodology, data curation, writing—original draft, writing—review, and editing. All authors contributed to the article and approved the submitted version.

## Funding

This study was supported by Leading House for the Latin American Region, the University of St. Gallen, Seed Money Grant, Switzerland.

## Conflict of Interest

The authors declare that the research was conducted in the absence of any commercial or financial relationships that could be construed as a potential conflict of interest.

## Publisher's Note

All claims expressed in this article are solely those of the authors and do not necessarily represent those of their affiliated organizations, or those of the publisher, the editors and the reviewers. Any product that may be evaluated in this article, or claim that may be made by its manufacturer, is not guaranteed or endorsed by the publisher.

## Footnotes

^1^The searches were carried out in two electronic databases: PubMed, and Google. The final search was performed on January 2022. The search terms and operators (AND, OR) used in the databases were: “data set” AND (biomechanics OR kinematics OR kinetics OR “inertial sensor” OR “wearable sensor” OR acceleration) AND Parkinson AND “Freezing of Gait”.
